# User perspectives on the future of mobility assistive devices: Understanding users’ assistive device experiences and needs

**DOI:** 10.1177/20556683221114790

**Published:** 2022-08-10

**Authors:** Leah Morris, Mary Cramp, Ailie Turton

**Affiliations:** School of Health and Wellbeing, 145292 UWE Bristol - Glenside Campus, Bristol, UK

**Keywords:** Impaired mobility, independent living, rehabilitation, stroke, amputation

## Abstract

**Introduction:**

Current assistive devices are inadequate in addressing the needs of some people living with impaired mobility. This study explored the experiences of living with impaired mobility in relation to how wearable assistive adaptive and rehabilitative technologies may improve their quality of life.

**Methods:**

A cross-case study approach was adopted; the case being defined as the experience of impaired mobility. Semi-structured interviews were utilised. The sample (*n* = 8) was purposefully selected to have impaired mobility due to stroke, age-related frailty, or lower limb amputation. From the interview transcripts, in-depth case illustrations were written to provide personal stories and thematic analysis was carried out to provide a cross-case analysis.

**Results:**

There were two overarching themes: lifestyle changes; and wishes and desires for assistive devices. There were shared experiences across participant groups, such as falls and fear of falling. All participants identified a wish for increased speed of walking. However, the reasons for their difficulties differed depending on personal factors and their condition. Participants wanted devices to be adjustable to their perceived ability on a day-to-day basis.

**Conclusions:**

Although common concerns and impacts of living with impaired mobility were apparent, individuals have unique requirements that should inform the design of assistive technology devices.

## Introduction

New ways of assisting mobility using wearable adaptive assistive rehabilitation technologies (AART) have the potential to improve quality of life for a growing number of people in the future. In the UK, 14.1 million people reported a disability in 2018/2019, with almost half of these disabilities being mobility-related.^1^ The leading causes of disability are musculoskeletal conditions and stroke, both of which are more prevalent in an older population.^2^ In 2018, there were 1.6 million people aged 85 years and over; this is predicted to increase to 3 million by 2043, resulting in a greater number of people living with mobility impairments.^3^ Furthermore, an increasing number of younger people are living with impaired mobility, due to rising prevalence of long term conditions.^4^

Commonly, those with mobility impairments use walking aids for assistance and to reduce their risk of falls.^5–7^ Those with lower-limb amputation commonly receive a prosthesis, as part of their rehabilitation process.^8^ These devices fall under the umbrella of ‘assistive technology’, which describes products or systems that assist those with disabilities, restricted mobility or other impairments to perform functions that might otherwise be challenging or impossible.^9^ Mobility assistive devices can in themselves cause challenges; for instance, users of four-wheel walkers have reported issues in opening doors and getting their walker onto public transport.^5^ Walking sticks limit a person to one free arm, while users of walkers without baskets have issues carrying items, food and drinks.^10^ In fact, the use of assistive devices can result in unsafe walking behaviours. An observational study of 16 participants found that they all used their walker incorrectly, this was particularly the case for front-wheeled walkers (a walker with two moving front wheels and two back ferrules) due to environmental factors (e.g., carpet edges) and design issues that hindered turning of the device, resulting in risky lifting strategies.^11^ Walking aid users have expressed concerns of becoming dependent on their walking aid, and the stigma attached to aids such as appearing old and bulky aids taking up space.^12^ A literature review on user perspectives of mobility assistive devices highlighted that the cost of these devices was also a key consideration.^13^ Evidently, current assistive and rehabilitative devices do not meet *every* user’s needs; however, AART developments may overcome these inadequacies.

Wearable powered, rigid exoskeletons have been offered as an alternative, with commercial devices in existence including the Ekso, Rewalk and Exo H2.^14–16^ Rigid exoskeletons have benefits such as increased walking distances, improved strength, and postural control in people with stroke. Nevertheless, exoskeletons have not been widely adopted clinically.^17^ It is not entirely evident *why*, however a systematic literature review on user perspectives of rigid exoskeletons has been carried out by.^18^ The review demonstrated some of the a range of limitations and concerns identified by patients and physiotherapist users of rigid exoskeletons, including: safety issues for example joint misalignment; challenges donning and doffing the device; cost; weight and device appearance.^16,19,20^

The developments in soft robotics may be able to address the limitations of rigid exoskeletons. A recent review on soft wearable robots reported rapid growth of using textiles/fabric-based actuators and pneumatic artificial muscles (PAMs), which are electrically-driven actuators.^21^ Bubble artificial muscles (BAMs) are one of the most lightweight pneumatic actuators that can be incorporated into a wearable exosuit and deliver high contraction or tension, to facilitate a person’s movement.^22^ These technologies have yet to be utilised to create a usable device for people with impaired mobility.

The soft-robotic garments should be more adaptable than current orthotics, and lighter and more comfortable than contemporary exoskeletons designed to assist mobility. Nonetheless, user wishes and evaluations of currently used hard exoskeletons illuminate some of the considerations for developing soft robotic AART. A survey of 354 wheelchair users demonstrated that minimising falls’ risk was the most important feature a exoskeleton could provide.^23^ Recent studies have explored users’ perspectives of exoskeletons for neurological rehabilitation, which highlighted the individuality of patient needs.^16,19,20^ For example, physiotherapists and patients did not like the set gait pattern of the exoskeleton, physiotherapists felt it imposed an unnatural gait pattern, and patients perceived it was doing all the work.^16^ If the device takes away user control and does not make the user aware of how it is facilitating their movement, it may not be effective for rehabilitation and may be rejected for use.^19,20^

For the development of future assistive devices, the experiences and wishes of those living with mobility impairments must be considered in the design process. The literature review by^18^ highlighted a limited number of studies integrating users within the design process of exoskeletons, inferring that this was due to the novelty and limited access of the technology. However, they drew upon wider evidence from more established assistive technology that suggests involving users in technology design ensures their complex needs are met.^18^ A study by^24^ explored the experiences of living with stroke and their use of technology, with the aim to provide technology developers an insight into values, thoughts, and feelings of potential users of robotic technology for rehabilitation of the hand and wrist. Participants offered rich qualitative descriptions of their wishes and needs in relation to their experiences of hand and wrist robotic technology. The study concluded that it is vital there is a clear understanding of how people with stroke make sense of their experiences and their perception of using technology.^24^ If the aim for future lower-limb assistive devices is to create devices that could have real impact on a user’s everyday life, then future research should follow the recommendations of preceding research.

### Objectives

This study was part of the Right Trousers research programme to realise a family of wearable, rehabilitative, soft robotic, AART devices for people who can walk, but who rely on assistive devices. The aims were to provide information about the experience of mobility impairment and wishes for future AART, so that those involved in device development understand the varied needs of potential users and appreciated the necessity to involve users in the design process.

## Methods

Favourable ethical opinion for the study was received in July 2015 from the University of the West of England’s Health and Applied Sciences Faculty Ethics Committee (UWE REC REF No: HAS 124 15/07/190).

### Design

The study utilised a cross-case study approach based upon the principles of Gerring,^25^ p.19) who specifies that ‘*a case may be created out of any phenomenon so long as it has identifiable boundaries and comprises the primary object of inference’*. Rather than focusing in on individual cases, cross-case studies focus on variation across individual cases. A clear phenomenon was set for this study: the experience of impaired mobility. See ‘Participants and recruitment’ section for the boundaries.

The method adopted was semi-structured interviews, a qualitative data collection strategy in which the researcher asks interviewees using a predetermined topic guide that has open-ended questions.^26^

### Participants and recruitment

Purposive sampling was adopted whereby the sample was selected based upon its ability to meet the study’s objectives.^27^ Participants were selected for one of three conditions: stroke, age-related frailty, and lower limb amputation. Age-related frailty was defined as a non-specific state of vulnerability, with changes being related to physical, psychological, cognitive and social factors .^28^ These three causes of impaired mobility were selected as the boundaries to provide a wide range of experiences so that engineers could appreciate common and differential requirements for AART. A secondary consideration was a selection of a similar number of male/female participants.

#### Inclusion criteria

Participants had to: be over 18 years of age; be able to give informed consent; have mobility impairments due to stroke, age-related frailty or lower limb amputation, and be able to walk but to have some self-declared impairment in their functional mobility.

#### Exclusion criteria

Individuals were excluded from participating if they had communication difficulties, aphasia, or inability to understand and express themselves confidently in English; communication or cognitive impairments would have prevented participants expressing themselves clearly and may have been distressing. To participate, individuals with stroke or amputation had to be at least 6 months post onset, to mitigate distress.

#### Recruitment

Recruitment was undertaken from October 2015 until June 2016. Support groups, charities and Public and Patient Engagement panels advertised the study including the researchers’ contact details. Researchers attended local groups to discuss the research, issue invitations and provide Participant Information Sheets. Individuals who were interested in participating could return a consent to contact form by post or email.

### Data collection

Eligible participants provided informed consent for the collection and use of data. The topic guides were based on three broad themes, the device-user perspective of living with impaired mobility; current assistive technology; and what users would most wish for from wearable AART. The topic guide was piloted with an interviewee known to the principal investigator, who commented on content and phrasing, resulting in a final guide (see Table 1).

**Table 1.** Topic guide.

A. What is the experience of mobility Loss in the lives of the interviewees
Background /context of lives
a. Family (prompts on relationships, any reliance)
b. Social (prompts on what they have enjoyed previously, now and what they would like to do)
c. Work - external and in the home
d. Using space (garden, garages etc.)
e. Family lives
B. What is the relationship between lives and devices
What are the existing supports or devices used/current health and social care solutions
a. [Relationships between living and the way they use their devices]
b. What are the limitations (linking back to background and using the prompts)
C**.** Needs and requirements from these assistive socks and trousers
Design/functions to be introduced to participants:
Device designed into socks or trousers
Sensors and actuators in a tight material
Assist with walking further, increasing pace
Assist with lifting foot
Assist with rising from a chair
Wish list for the socks and trousers – what does the participant want them to be able to do and what are the key design considerations? (link back A and B above)

Interviews were undertaken by two researchers, a post-doctoral researcher (SM) with qualitative research expertise, and an academic Occupational Therapist (author, AT). AT had clinical experience with people with impaired mobility due to stroke, amputation, and age-related musculoskeletal conditions. The differing backgrounds led to variation in interviewing styles and subjectivities enabling a broad spectrum of information to be captured.^29,30^ At the time of the study, AT was a project co-applicant and SM a Research Fellow. Neither researcher knew the participants prior to the study. Interviews were undertaken in people’s homes and only participants and researchers were present during the interviews.

SM made the interview arrangements and informed the participant that she would be carrying out the interview with another member of the research team. Participants were informed on the aims of the study, who would be interviewing them, and that AT was an Occupational Therapist.

On arrival, SM introduced both researchers and explained that she would ask most of the questions, but that the co-researcher (AT) would ask follow-up questions, to obtain a detailed picture of the participant’s experiences and views. Although AT may have had some biases when interviewing due to her occupational therapy experience, the effect on the interview is most likely limited as she was only second interviewer. SM did not report any conscious biases. Interviews were audio recorded on a Dictaphone and field notes taken during the conversation. Questions followed the topic guide but were tailored to the interviewee with probes used to clarify statements that may be ambiguous or contradictory.

Field notes were left as raw data as a case record. Recorded audio interviews were transcribed, and verbatim transcriptions were uploaded to a secure online storage system and were uploaded into NVIVO 12. Summaries of the participant’s experience of living with impaired mobility were developed as personal stories and they were sent to each participant for corrections or comment. This provided a check for accuracy and corroborated findings .^29,31^ The individual case illustrations for the case study are available at UWE repository [https://uwe-repository.worktribe.com/output/7278673].

### Data analysis

For the cross case analysis, to determine commonalities and differences between the individuals’ experiences, interviews were analysed through thematic analysis.^32^ A researcher, who was not present at the interviews (LM) coded using a semantic approach; codes and themes are identified within the explicit meanings of the data and the analyst is not looking for anything beyond what a participant has said. Codes were created explicitly from the extracted data. Themes were then created; a theme captured something important about the data in relation to the topic and represented some meaning within the data set.^32^ As the aim of the study was to gather an in-depth case study of impaired mobility, it was not necessary to demonstrate data saturation.^33^

Similar codes were amalgamated into overarching themes. Themes were reviewed by reworking data extracts that did not fit and altering theme titles as needed. To manage the large coding set, the themes were broken down into subthemes.^32^ A consideration when forming the narrative was a representation of data from across all the participants.

## Results

### Sample

Table 2 gives the characteristics of the interview participants. To summarise, there were 8 participants in total, ranging from 48 to 89 years old, and five were male. Participants had either previously had a stroke (*n* = 3), were older with aged-related frailty (*n* = 2), or had a prosthesis due to amputation (*n* = 3). Participants referenced a range of walking aids they used, including walking sticks, walking frames; crutches, and walkers and foot-ups (soft elastic orthotics to lift the toe and front part of the foot). They used a range of home supports, including stair lifts, perching stools, wheelchairs, stair bannisters, bathroom grab rails, support from others and furniture to help with walking.

**Table 2.** Participants.

Code	Gender	Age	Health information	Devices used
Alex	Male	61	6 years post-stroke	Walking stick; soft elastic ‘Foot up’ orthotic for assisting with dropped foot; hand rails on the stairs
Paul	Male	52	2 years post-stroke	Walking stick for outside; perching stool; ground floor accommodation; wet room; grab rails
Dianne	Female	48	3 years post-stroke	Walking stick for outside; walker with a seat; mobility scooter long distances; hand-rails on stairs; seat on the bath; shower stool; grab rails
Gwen	Female	89	2 years living in a residential home due to age-related frailty	Walker with sliders (like short skis); wheelchair
Francis	Male	>80	4 weeks living in a residential home due to age-related frailty	2 walking sticks; rollator (a walker with wheels)
Peter	Male	50	2 years living with a left leg knee disarticulation amputation	Two different types of prosthetic legs manual wheelchair; crutches; walking stick; handrails bathroom; seat across the bath; bungalow – no stairs or steps
Tony	Male	67	42 years living with a left below knee amputation	Two prosthetic legs; crutches; walking sticks; house with stairs
Sheila	Female	61	6 years living with a left below knee amputation	Two prosthetic legs; walking frames crutches; manual wheelchair; perching stool; house with stair lift; downstairs bathroom

Interview length ranged from 30 min to 1 h 24 min. All participants approved of their case illustrations and no new materials arose from the respondent validation ^31^

There were two overarching themes: (1) ‘Lifestyle changes’ and (2) ‘Wishes for new assistive technology’ (see Table 3 for themes). This paper aimed to connect the concepts of user’s experience with their wishes for future assistive devices. Theme (1) provided a rich personal background to increase the reader’s understanding of the impact of impaired mobility on these individuals. Theme (2) integrated participants’ experiences of current assistive devices and their wider experiences of impairment with their wishes for future devices. We recognise that although there are similar themes across the three participant groups, the cause of their difficulties are different, and thus how devices address their wishes and desires will differ. As such, sub-headings highlight sub-themes that are population-specific. Statements are clearly linked to the underlying impairment.

**Table 3.** Themes.

Lifestyle changes	Wishes and desires for assistive devices
• Participation	• Improving distance and pace
• Adaptations around the house	• Holistic device
• Work	• Prevention of falls
• Driving	• Increased foot sensation and control
• Forward planning	• Required assistance and power
	• Ease of dressing
	• Material properties

## Theme 1 – Lifestyle changes

### Subtheme 1.1 – Participation: All participant groups

All the participants highlighted activities that they used to partake in but could no longer, including long walks with others, exercising in the gym, cycling, rugby, organising community activities, and fishing. Curtailment of activities were due to fatigue, and, for the amputee group, the limitations of the socket fit of their prostheses which resulted in the leg becoming unattached, causing embarrassment. Peter, a male amputee participant, and Paul, a male stroke participant, discussed others perceiving them as drunk due their abnormal gait:

*“My walking went completely off kilter and people were looking at me as if I was… there were some strange looks on their faces, almost like ‘what is wrong with you’ kind of thing.”* (Paul)

Fear of falling affected participation, as it prevented an older female (Gwen) from walking outdoors without assistance of others, and two male amputee participants (Tony and Peter) and a female stroke participant (Dianne) would not go out in poor weather conditions. A male amputee and male stroke participant stated that to do the things that were important to them, they had to overcome this fear:

“I got very, very stressed on the train coming back because I don’t know if you’ve ever noticed, I hadn’t noticed before, the gap getting off the train at *train station* is enormous” … “I’m a lot better now because I had some hypnotherapy this year and that’s made me a lot more confident.” (Alex)

*“I went to a castle a while back and went up the stairs, and it was a tight twisting staircase and all the rest, and I got up, and I got back down, but I was petrified going up and petrified coming down, but I was going to do it because I wanted to do it.”* (Peter)

### Subtheme 1.2 – Adaptations around the house: Amputee and stroke participants

To manage their fatigue participants sat down more frequently whilst doing housework.

#### Adaptations around the house: Amputee participant – washing with no lower limb sensation

All amputee and all stroke participants discussed altering the way they washed due to the risk of slipping; a male amputee (Peter) no longer showered as he could not feel the shower floor:

*“Even with this [prosthetic] I wouldn’t like to do it [shower], because you can’t feel the bottom of it, it’s very easy to slip” (*Peter)

#### Adaptations around the house: Stroke participants – limited strength when washing

A stroke participant (Diane) had grabrails put in her shower. Alex, a stroke participant, could not lift his leg to get into the bath and so only had showers:

*“One of my great pleasures in life was lying in a bath, soaking in a bath listening to sport on radio, and I haven’t done that for six years.” … “I worked at it with my physio, hip hitching for ages and basically gave up, I just couldn’t do it. It was actually a knee bend that I couldn’t do.”* (Alex)

### Subtheme 1.3 - Work: Amputee and stroke participants

Some individuals maintained working roles after the onset of their condition, but predominantly the working lives of working age interviewees had diminished. For amputee participants Sheila and Tony, their impairment forced them to give up work:

*“I mean the doctor sys well just retire on ill health. It was getting a long journey for me to go to work every day”* (Sheila)

For stroke participant, Alex, his work was directly impacted by impaired mobility; he continued in work roles despite issues commuting via trains and difficulties in building access that resulted in a new pavement being built:

*“The pathway up to the office used to be dangerous anyway. The pavement was really rubbish.”* (Alex)

### Subtheme 1.4 – Driving: Stroke and amputee participants

There were issues for a stroke (Paul) and amputee participant (Peter) in transferring into cars:

*“they’ve [friends] got a sports car, and it’s nice and it’s* good*, but I have to cling onto the roof to lower myself in, and it’s the same getting out the other end, there is physically no way I can do it without climbing out using my arms.”* (Peter)

*“Getting in and out of cars I find difficult because obviously being in this country, because you drive on the right-hand side, it feels like I have to try and get in the car with the left leg. So, what I tend to do is to go in sideways and sort of swing my legs round.”* (Paul)

Two amputees (Peter, Tony) and a stroke (Alex) participant highlighted switching to an automatic drive due to lack of clutch control. For Alex, this had a large impact as he was forced to sell his prized car.

### Subtheme 1.5 – Forward planning: Stroke and amputee participants

A new aspect of life for participants was the constant need for planning. An amputee participant (Peter) expressed being unable to spontaneously run to catch up with his young daughter, as he had to plan which prosthesis he wore for different activities. He also expressed limitations towards sexual intimacy with his partner, as his limited control of the prosthesis required removal in advance. An amputee participant (Sheila) and two stroke participants (Paul, Alex) expressed meticulous planning when going to new places due to access requirements:

*“I have to plan in extreme detail where I’m going to park the car because I have to know that there will be somewhere to park the car that won’t involve me crossing the road because I only cross roads at pedestrian crossings. I need to cross roads where there’s a flat surface.”* (Alex)

## Theme 2 – Wishes and desires for assistive devices

### Subtheme 2.1 Improving distance and pace: Stroke and amputee participants

Participants expressed that their pace and distance walking had reduced. Two in the amputee group (Sheila, Peter) and all the participants in the stroke group worried about being a burden when they were not able to keep pace with others when walking:

*“If I could walk better, faster” … “Because I wouldn’t rely on other people to wait for me, I can walk at their pace and not my pace, that would be nice.”* (Dianne)

#### Improving distance and pace: Amputee participant stump changing shape

Specific to amputee participants was the desire for a device that could remove the need for a liner and could mould around their stump, as they had issues with the stump changing shape when walking long distances (Peter, Sheila):

*“If I was to go for a longish walk as well, that’s another big problem. Your stump changes shape, if it’s cold it shrinks and then nothing fits properly, and that’s when you start getting problems.”* (Peter)

### Subtheme 2.2. - Holistic device: All participant groups

Participants frequently had more than one type of walking aid and it was evident that there was no ‘perfect’ walking aid or prosthesis for all environments. For shorter distances, a female stroke (Dianne) and older female participant (Gwen) chose their walking stick, but when walking any distance outdoors they chose their wheeled walker. When asked why, the stroke participant answered:

*“Probably the distance, and also if I need to sit down and have a rest I can sit on the walker and rest.”* (Dianne)

The space that the aid/support took up was a consideration for some (Francis, Dianne, Sheila), as it was perceived they could *“clutter up the room”* (Francis, older person)*.* Sheila highlighted that having a range of prosthetics in her room acted as a constant reminder of what she had lost:

*“It's the first thing you see when you...well it isn't, it's your missing leg that you see first. I think you’ve got to have all this; it brings it home.”* (Sheila, amputee participant)

#### Holistic device: Amputee participants - trade-offs made

A male amputee participant highlighted the trade-off made when selecting a prosthesis, as his lighter prosthesis did not have stumble recovery and he was more likely to trip:

*“I have got another leg, I’ve got a Total Knee 2000 which is very easy to use, you can whizz along with that thing much easier, and it’s probably about half the weight I imagine, unfortunately it’s got no stumble recovery whatsoever.”* (Peter)

However, he highlighted that there was lack of control when using the stumble recovery prosthetic limb. This resulted in him falling backwards when sitting in low chairs or sitting on the floor to play with his daughter, and he was fearful of kicking people in the process. All the amputee participants highlighted weight restrictions of the prosthesis.

These findings highlight that distance and pace are important considerations for assistive device users, but there are other considerations including the space they take up and stumble recovery.

### Subtheme 2.3 - Prevention of falls: All participant groups

Loss of balance was a cause of falls across the participants, with reference to challenges balancing whilst toileting (Gwen, Dianne), and issues outdoors due to uneven terrain and icy or wet weather (experienced by all stroke and all older participants). Stroke participants had experienced falls due to reduced strength, impaired sensation, and lack of ability to lift the foot.

A stroke participant (Dianne) and amputee participant (Peter) expressed the wish for future devices to increase their confidence through prevention of falls:

*‘Oh, if it could make a difference to me doing… if I could do kerbs on the road and walk further, that would be a huge step forward’* (Dianne)

*‘If you ever got something that could in effect stop you falling over, or at least stop you falling over any more than anybody else, I would have thought that would then boost their confidence which would then increase the amount of time they’re prepared to spend walking’* (Peter)

### Subtheme 2.3: Increased foot sensation and control: Amputee and stroke participants

#### Increased foot sensation and control: Stroke participant - lifting his foot

Two stroke participants had difficulty lifting their affected foot, and this specific need meant they valued a device providing foot control:

*“Because what I found is that if I am distracted by something, it is usually when it [foot] stops working. So, if I am thinking about other processes, so something that could sort of takeover that role instead of me having to concentrate on it all of the time.”* (Paul)

#### Increased foot sensation and control: Amputee participant – lack of feeling in his leg

Similarly, an amputee participant shared an experience that resulted in him wishing for a device which could increase feeling in their leg and thus their control:

*“Just to have that feeling of… have it feeling less like a dead leg, if you like. So being more proactive, that would possibly be a nice feeling.”* (Peter)

### Subtheme 2.4: Required assistance and power: All participant groups

Participants were questioned on where it might be acceptable to carry the power pack, in a backpack or on a belt. A belt appeared to be most acceptable to all the stroke participants, with one participant stating that he would not want a backpack as he wanted something:

*“That you actually didn’t have to think about, because a pack would actually be limiting in other ways it seems to me.”* (Alex)

The value of the activity or function that the device could provide dictated the acceptability of the weight of the power supply for the device:

*“You will probably reach a point with the weight of the battery where it is not worth the carry if you see what I mean.”* (Alex)

#### Required assistance and power: Amputee and stroke participants want to be active

When discussing assistance from walking aids, a stroke participant (Paul) highlighted concerns of: *“getting overly reliant on it [a stick]”* as he was worried that the stick would reduce his strength on his affected stroke side. An amputee participant similarly expressed initial reluctance to use his prosthesis, however, gradually he accepted his reliance on it:

*“But gradually as you start to use it better and you get better with it, it sort of becomes something you rely on, and I actually quite like it now.”* (Peter)

When discussing future assistive devices, an older male participant highlighted that the required power or assistance may change as his mobility deteriorated:

*“Well, I feel I’m at the stage where I don’t need that actual help, but I’ve got to be sensible that in six- or twelve-months’ time I might need some assistance of that type.”* (Francis)

#### Required assistance and power: Older persons become passive

While the stroke participants and amputee participants had wanted to hold onto their independence, the older group highlighted a passivity to the help of carers. The participants living in residential care homes (Gwen, Francis) discussed the carers assistance getting them out of bed, dressing, and transferring to the dining room. They acknowledged that their mobility had deteriorated:

*“I was quite capable, but I’ve been here two years now and this is where I’m finding the difficulty.”* (Gwen)

#### Required assistance and power: Amputee and stroke participants’ day-to-day mobility variability

It should be noted that the required assistance of individuals varied day-to-day. Two stroke participants (Paul, Dianne) and two older participants discussed the variability of their mobility:

*“Yes, I tend to push up through from my right-hand side anyway, to start off. When I have a bad day, I have to sort of get the leg to swing to get it to start moving.”* (Paul)

Similarly, an older participant (Gwen) expressed needing help from carers to swing her legs over the bed on days where she was low in energy. When a stroke participant was asked how she decided what she would wear or what walking aid she would use, she stated it was contingent on how she was feeling that day:

*“I just wait, I just like to wake up and see if I feel any different, see what I’m going to wear and use that day.”* (Dianne)

### Subtheme 2.5 - Ease of dressing: All participant groups

Although individuals had their own rationale, it was universal across participants that they wanted the device to be easy to put on. An older participant (Gwen) expressed issues bending down to get dressed, an amputee participant (Tony) highlighted issues of trousers catching on the prosthesis as well as the requirement for easy access to his prosthetic. An amputee participant highlighted that over time his ability to dress himself may deteriorate:

*“Yes, it’s all very well having something that I can put on at this particular point in my life, whereas in a few years’ time I’m physically incapable of doing it.”* (Peter)

#### Ease of dressing: Stroke participants limited to use of one arm

Two stroke participants (Paul, Dianne) discussed challenges dressing with limited use of their arm affected by stroke, and they highlighted the inability to use zips or buttons and thus the need for elasticated waisted trousers:

*“Because when I put the sleeves on my leg, I have to put it on and then sort of pull it up with my right hand because I can’t grip it enough with my left hand to get it up and over. Because they are quite tight as well – obviously – especially getting up and over the last bit, over the* foot *and the bit around the ankle.”* (Paul)

For one stroke participant (Dianne), dressing was such a long and tiring process that assisting with dressing was the key activity that she wanted the device help with.

#### Ease of dressing: Older persons desired long-wear

Two older participants (Gwen, Francis) highlighted that it was the carers who dressed them; consequently, it was more important that the device could be worn all day, rather than just for the duration of activities:

*Q: “If you wore them would you wear them for say an hour a day to help you walk up and down the corridor a bit?”*

*A: “Leave them on all day until I go to bed.”*

*Q “You’d leave them on all day, yeah. Because once they’re on, they’re on.”*

*A “They’re on, yeah. But the difficulty is getting them on because the girls do that.”* (Gwen)

#### Ease of dressing: Amputee and stroke participants – device appearance

It was important for there to be a choice in the garment, with tights being preferable to a female amputee participant who discussed not feeling feminine in trousers. All the stroke participants expressed the want for the device to be discreet, with a neutral colour expressed as preferable:

“*I think it would be important it [the device] didn’t show in a way’… ‘I am trying to think for myself, because I know some people would actually object to wearing something that made them look different.”* (Alex)

However, one amputee participant explained how he wore shorts so that people did know he had a disability, highlighting the variety of preferences for device appearance:

*“Two reasons: one is they’re a lot more comfortable, and number two is, it stops people thinking I’m drunk.”* (Peter)

### Subtheme 2.6 - Material properties: All participant groups

A participant from each group highlighted the wish for the material to have a comfortable feel (Francis, Sheila, Paul). It was desirable to a participant in each group (Sheila, Paul, Gwen) for the material to be thermal, due to personal fluctuating temperatures and seasonal changes.

## Discussion

This cross-case study illuminates how participants’ experience of impaired mobility affected their lives. Participants experienced reduced speed and distance in walking, and increased effort needed to walk and get up from sitting, all of which constrained their daily activities. Fears of falling were common and due to anxieties about slipping and increased reliance on others, participants limited their excursions outside the home. Changes in lifestyle also resulted from social embarrassment, for example, from others having to wait for them. The case illustrations (see https://uwe-repository.worktribe.com/output/7278673) highlighted individual desires for solutions to reduce the difficulties experienced because of personal factors. The cross-case analysis showed some common wishes participants had for improving their everyday lives.

Vitally, participants’ concerns for future wearable devices were that they should be easy to put on and comfortable to wear for the whole day, as participants already found it challenging to dress. A wish for the device to be discreet was expressed, which may be best understood alongside the ‘Participation’ theme, which highlighted embarrassment due to their disability. A similar finding was present in Ref. 34 in which users found mobility devices as stigmatizing and therefore they were unhappy using them in public. Additionally, our findings highlighted the importance of using comfortable materials for the device. Several of these priorities were apparent in a survey of wheelchair users who were asked about their requirements for exoskeleton technology.^23^ Participants ranked the importance of 17 properties of the exoskeleton; comfort was ranked 3rd and ease of dressing was ranked 5th, while in the sample, pace and appearance had limited importance (15 and 17 respectively).^23^ However, the priorities of participants in that study may have been different as they had less residual mobility and were dependent on using a wheelchair. Whereas prosthesis users’ highlighted the importance of both comfort and appearance of the prosthesis.^35^ This indicates that the needs of one user may not be identical to the needs of another, therefore a range of contexts must be considered when designing several assistive devices.

Our study was part of a research programme aiming to develop soft robotic garments for assisting mobility and rehabilitation in people who can walk, but whose mobility is impaired [www.therighttrousers.com]. It can be inferred from the findings of this cross-case analysis that an assistive device providing too much assistance than is required could be detrimental to the user’s mobility; this was highlighted by the ‘Required assistance and power’ theme. Further, the required assistance power varied for all groups, suggesting that adjustable or adaptable assistance is desirable in wearable mobility devices, to cater for variation in performance either in rehabilitation or in day-to-day health.

Considering the ‘Wishes and desires for assistive devices’ is important, but nonetheless, presenting these findings does not mean that they are achievable in device design – or at least in the immediate future. There are trade-offs and disadvantages that will have to be tolerated while wearable assistive devices are developed. Key themes within this paper included the desire to walk further and faster, but also that the device could become unacceptable if it was too heavy. Other studies have reported similar findings, for example, an exosuit made from textiles, mechanical actuators and other components can improve the speed and distance for walking in stroke patients.^36^ However, the exosuit required the user to carry a 4.6 kg load, thus limiting the pool of stroke survivors who could use it. A review of 52 exoskeletons found that, as well as on-board actuators having drawbacks in regards to weight, the devices with off-board air supplies were also restrictive for ambulation.^37^ Further, the review highlighted that only two of the 52 exoskeletons included a combination of soft structure and compliant actuation (as opposed to rigid, heavy actuators) and recommended further development of lightweight devices. That review found that there was limited evaluation of the user’s perception of the exoskeleton for all 52 exoskeletons.^37^ However, engaging potential users and stakeholders throughout the design processes are important for successful adoption.^38^

This paper and wider evidence highlights that, to achieve useful wearable assistive technology for improving mobility in everyday living, designers and engineers need to consider much more than biomechanics, actuator assistance, sensors and control processes to move limb segments. While other studies focus only on the experiences of those with impaired mobility,^6,34^ the novelty of this paper is its ability to connect these experiences with their future wishes for lower-limb assistive devices. A similar study has been carried out by^24^ which explored the experience of living with stroke and using technology, however, the assistive device was for the upper limb, not an exoskeleton or wearable mobility device. Nevertheless, the study highlighted the great potential to include participants in the design process, including user’s qualitative evaluation of prototypes. A literature review of stakeholder perspectives on mobility assistive technology highlighted a consensus on the need for future research focusing on the user’s active involvement in the AART design process.^13^

Ways of actively involving users in technology design processes are continuing to evolve with co-production encouraged. We recommend that future research to develop AART involves close partnership with device users and an interactive approach. The current study has informed the approach taken within the Freehab study (www.therighttrousers.com), a continuation of the Right Trousers project, that looks to design a soft-robotic, wearable, lower-limb assistive device. Physiotherapists and a patient partner have been involved in inter-disciplinary meetings where they have contributed to discussions about how and why facilitation is provided during rehabilitation and, through seeing early prototypes, to the technological developments. We have found that health researchers have been able to provide the bridge between user partners and engineers, to ensure a shared language and understanding when discussing devices. Although still early in our developments, the team have found users involvement essential in providing direction for design. However, there are challenges to the process, such as organising dates to suit busy healthcare professionals, patient partners and researchers. The process is an active learning process, and we intend to publish recommendations from our experiences in the future.

The findings of this paper highlighted that despite there being similarities within participant groups, there were variations with subthemes that will greatly affect how a device may assist that patient. For example, both the stroke and amputee groups wanted a device to facilitate foot clearance when they are walking. However, the reasoning for needing facilitation differed; stroke participants highlighted the issue of having to concentrate on clearing the foot of the ground, while an amputee participant highlighted that their issue was due to having no feeling in one leg. Clearly an assistive device would not be able to use an identical solution for both these groups to clear their foot of the ground. Therefore, when designing devices, engineers must decide whether to design devices for specific pathologies/cause of impairments, or alternatively, design a greater number of devices that address common wishes for devices but with different solutions. Similar findings are present in Ref. 16, as they concluded that it was essential to define client selection criteria for exoskeletons so that it was clear who could benefit from the technology.

There are recognised limitations of this study. While the diversity of medical conditions was deliberate to inform engineers of a breadth of experiences, the sample size is small (*n* = 8), and so transferability of findings to populations is limited. However, participants were purposefully selected to provide in-depth narratives of their experiences of living with impaired mobility, similar to the rationale of ^24^ for their sample size of 10 PWS. For this paper, we asked feedback from the engineers on the value of the case studies, with all five respondents stating that they were useful, and they discussed how they might incorporate the findings into design devices in the future. This feedback demonstrates the worthwhile nature of case studies as a method. It is available at: https://uwe-repository.worktribe.com/output/7278673. Interviews were not coded by the interviewers which can influence the credibility of the findings, as without forming relationships with interviewees and in the absence of visual cues during the interview, the coder may have interpreted participant’s meanings differently to their intention.^39^ However, some mitigation of this risk was given by checking with an investigator who was present at the interviews.

### Conclusion

We hope that the cross-case findings and the personal stories provided by participants will help engineers to understand the many and varied problems that need to be addressed in designing wearable devices for improving mobility. Participants were interested in the prospect of wearable soft robotic garment to improve their mobility. Their experiences serve to illustrate the importance of considering context in designing wearable devices for improving mobility, which has historically been absent form exoskeleton research. Involving potential users in co-design should improve the chances of successful device development and adoption.
